# Round the Hospitals

**Published:** 1921-06-11

**Authors:** 


### ROUND THE HOSPITALS.

The visit of the Queen to the Salvation Army Mothers' Hospital at Clapton brings into prominence the large part taken by nurses in this world-wide organisation. The new Nurses' Home, inspected by Her Majesty with her accustomed thoroughness and sympathetic insight into details affecting the comfort of nurses, has been admirably planned to give separate bedrooms prettily furnished and fitted with gas-fires to accommodate twenty-four nurses, with good community-rooms and modern offices. A debt of £5,000 remains on it to be discharged by the public, and further efforts are required to complete the hospital by adding more wards and rebuilding the frontage. An average of 1,000 births take place annually within the wards, where seventy-two beds are available. The occasion of the Queen's visit was rendered unique by the presence of many representatives of the British Dominions and Allied nations now visiting London to attend the International Social Council. Ten representative officers were presented to Her Majesty, including Commissioners Adelaide Cox and Mildred Duff, Superintendents of the rescue-work, and Lieut.-Colonel Miriam Castle, the fully-trained and certificated Matron of the hospital. Officers from the United States, Canada, India, South Africa, Australia, the West Indies, and Russia were also presented.

---

Birmingham has risen enthusiastically to the call from the nurses, and the Bingley Hall Scenic Fair is admittedly the most admirably organised which the city has ever seen. Princess Mary, who was received by a body-guard of nurses in uniform, was present on June 6 and received nurses. Twenty-eight matrons of hospitals assisting at the Fair, whose names appear on another page, were presented to Her Royal Highness, who appeared greatly to enjoy the animated scene. On the following day the Fair was opened by Viscountess Cowdray, whom Sir Arthur Stanley described as the best friend nurses have ever had or would have. The guard of honour on this occasion was formed by the members of the College. Almost every town and hamlet in Warwickshire, Worcestershire, and Staffordshire has had its share in producing the wonderful effect. The Nurses' Club for which this splendid effort was made is assured beyond all question, and the College of Nursing and the Nation's Fund will benefit by substantial grants. Best of all, perhaps, the three counties have learned throughout their length and breadth something of the enthusiasm, the live progressive spirit, and the public aims of the nurses who dwell within their borders.

---

The opening of the new headquarters of the General Nursing Council for England and Wales at 12 York Gate, Regent's Park, which after June 8 will be the Council's only address, takes place on June 10, at 3.30, as this number of The Hospital appears. It will be a striking occasion, and the choice of Princess Christian, whose name has so long been identified with the cause of State Registration, to preside at the opening ceremony was very appropriate. The Chairmen and Matrons of all the large voluntary hospitals and Poor-law Infirmaries are invited to be present, and representatives are also invited from the London County Council, Metropolitan Asylums Board, and nursing associations.

---

It is proposed to hold a Masque and Pageant in Norwich later in the summer under the direction of Mr. Nugent Monck, in aid of the Nurse Cavell District Nurses and All Hallows Sisters. Nurses and others willing to take part in the pageant are requested to communicate with the Hon. Secretaries, The Crypt, Ninham's Court, Norwich. At the recent annual meeting of the District Nurses Association more than one speaker spoke of the high sense of duty shown by the nurses and of the love and gratitude felt for them by their patients. One-fifth of the sum contributed comes from patients.

---

The Great Northern Central Hospital is one of the most active of Metropolitan hospitals, and it is not surprising that the bazaar at Crewe House on Thursday and Friday, June 2 and 3, attracted a numerous and eager band of buyers who continuously poured through the beautiful rooms intent on purchasing. The excellently arranged and printed "Guide to the Bazaar" contributed greatly to the comfort of buyers. Unlike many bazaars the prices of the articles sold were not only for the rich; many bargains were rapturously exhibited by the pleased purchasers, whilst the quality and variety of the goods sold at the various stalls was excellent. One of the most interesting stalls was that of the Disabled Soldiers, for the sale of articles made entirely by one-armed men at the Lord Roberts Memorial Workshops Training Centre, London. The Marquis of Northampton, chairman of the hospital, in his speech at the opening, stressed the fact that the Great Northern Central Hospital is the only general hospital in North London, that though in debt to its bankers and others to the extent of about £30,000 it has not closed any of its wards, that it was the first hospital to start contributory wards, that it has established an insurance scheme for patients, and that it works in co-ordination with the small hospitals and institutions for the sick in the area it serves; he also claimed special consideration in view of the fact that the cost per bed, £143, of the Great Northern Central Hospital is below the average of the bed cost of sixteen of the largest hospitals, viz. £155, but he did not mention that amongst these, presumably, the large teaching hospitals with medical schools are included. Princess Alice, Countess of Athlone, opened the bazaar in a charming and effective short speech, in which she appealed for the heads, hearts and hands of her audience on behalf of the hospital. We are glad to know that the

**ROUND THE HOSPITALS**—(*continued*).

financial results of the bazaar up to the present are highly gratifying and already exceed the anticipated total of £2,000.

In his letter of March 21 (The Hospital of March 26, page 592), Mr. W. R. L. Blakiston, writing on behalf of the Minister of Labour to announce that a probationer and a nurse employed at a voluntary hospital to nurse patients are required to be insured under the Unemployment Insurance Act, stated that the position with regard to sisters at a voluntary hospital was still under consideration and a further communication would be made on that question. We are now informed a decision will shortly be given. At the same time the official pronouncement is that the employment of nursing sisters is covered in principle by the decision given in respect to nurses above referred to; sisters are not regarded as employed in domestic service, and pending further decision are required to be insured subject to the general provisions of the Act. The general principle is the same under the Unemployment Insurance Act as under the National Health Insurance Acts, and unemployment insurance contributions are payable in respect of any persons for whom health insurance contributions are payable.

A large group of district nurses, many with war decorations, with a party of nurses from St. Mary's Hospital, Plaistow, assembled at the opening of the Red Triangle Club to welcome the King and Queen as they passed through the women's department. Their share in the numerous activities represented is by no means a small one.

On the occasion of Princess Mary's visit to Birmingham she paid a visit to the Infant Welfare Centre at Floodgate Street, the first institution of its kind to be opened in this country. Dr. Robertson, the Medical Officer of Health, and Miss Peace, the Lady Superintendent, were presented to H.R.H.

The vacancy for a lady superintendent of the Nurses' Co-operation, caused by the resignation of Miss Hoadley, has been filled by the appointment of Miss J. M. Jackson, O.B.E., R.R.C., at present matron of the New End Hospital, Hampstead, which post she has held since 1920. Miss Jackson, who was trained at St. Bartholomew's Hospital and the Rotunda Hospital, Dublin, has had considerable and varied experience in the nursing world, which should stand her in good stead in dealing with the many types and classes of people whom the Superintendent of the largest Co-operation of nurses must be prepared to encounter. Successively ward sister at the New Hospital for Women, Euston Road, assistant matron at Kensington Infirmary, assistant matron at the Royal Sussex County Hospital, Brighton, matron of the Royal Surrey County Hospital, where, in the matron's absence, she was responsible for the private nursing staff, she was called up in Queen Alexandra's Imperial Military Nursing Service Reserve and served during the war as matron, first in Egypt and later in India. She was subsequently lent to the South African Hospital, Richmond, where she filled the office of matron with much success. Miss Jackson will take up her duties about the middle of July.

The directors of the Arbroath Infirmary have decided to refuse the offer by the Town Council and Red Cross Society of Greenbank House to be used as the Nurses' Home in connection with the Infirmary. General regret was experienced that they could not see their way to accept the offer, and a committee has been formed to go into the matter and remove possible objections. There is a sum of £2,800 in the hands of the Red Cross Society, part of which it was proposed should be used for putting the house in good order for the nurses and the rest invested for the use of the Infirmary.

The Steyning Guardians are faced with a complete *impasse* in the case of the two probationers who after passing their final examination were refused the usual certificate on account of insubordination towards their superior officers and unkindness towards patients. It will be remembered that the Board decided to ask Dr. Kinloch, who conducted the examination, to sign the nurses' certificate. This he has absolutely declined to do on learning that Dr. Allfrey, the medical officer, had declined to do so, and that Miss Carter, the superintendent nurse, had declined to recommend the nurses for any certificate. A prolonged discussion on the case took place at the last meeting of the board, and a good deal of evidence from patients and from other nurses bearing against the probationers was alluded to. The Inspector of the Ministry of Health declares the Guardians are in the hands of the doctor, who cannot be forced to sign the certificates. It was eventually decided by seventeen votes to seven to lay the whole of the facts before the Ministry of Health and leave the matter to their decision.

The result of Pound Day at the Royal Salop Infirmary was most gratifying, the matron receiving about 1,283 pounds of groceries, etc., and cash to the amount of £115 17*s*. in lieu of gifts in kind. This institution has an excellent system of parish hampers—most of the neighbouring villages undertaking to fill one or two hampers each year with farm or garden produce. Nearly all the fruit and vegetables used in the Infirmary are provided. For the last seven years an average of over 20,000 eggs has been received, so that this item does not appear in the hospital accounts. The value of Pound Days is twofold. Not only do they save the hospital's purse, but they are an incalculable asset in the vital interest which is thus stimulated in the surrounding districts on behalf of the hospital as an institution. Such interest once kindled and encouraged does not easily die.

